# The suppression of fibroblast growth factor 2/fibroblast growth factor 4-dependent tumour angiogenesis and growth by the anti-growth factor activity of dextran derivative (CMDB7).

**DOI:** 10.1038/bjc.1998.451

**Published:** 1998-07

**Authors:** R. Bagheri-Yarmand, Y. Kourbali, C. Mabilat, J. F. Morère, A. Martin, H. Lu, C. Soria, J. Jozefonvicz, M. Crépin

**Affiliations:** Laboratoire d'Oncologie Moléculaire Humaine (EA 445), UFR Léonard de Vinci, Université Paris-Nord, Bobigny, France.

## Abstract

**Images:**


British Journal of Cancer (1998) 78(1), 111–118
© 1998 Cancer Research Campaign

### The suppression of fibroblast growth factor 2/fibroblast growth factor 4-dependent tumour angiogenesis and growth by the anti-growth factor activity of dextran derivative (CMDB7)

**R Bagheri-Yarmand[1], Y Kourbali[1], C Mabilat[2], JF Morère[3], A Martin[4], H Lu[2], C Soria[2], J Jozefonvicz[5] and M Crépin[1]**

[1]Laboratoire d'Oncologie Moléculaire Humaine (EA 445), UFR Léonard de Vinci, Université Paris-Nord, 74 Rue Marcel Cachin, 93017 Bobigny cedex, France; [2]Inserm U353, Institut d'Hématologie, Hôpital Saint Louis, Paris; [3]Groupe Nord d'Oncologie multidisciplinaire and [4]Service d'Anatomie Pathologie, 125 avenue de Stalingrad, 93000 Bobigny; [5]Laboratoire de Recherches sur les Macromolécules, CNRS, URA 502, Université Paris-Nord, Avenue JB Clément, 93430 Villetaneuse, France

**Summary** Our previous studies showed that carboxymethyl benzylamide dextran (CMDB7) blocks basic fibroblast growth factor (FGF-2)-dependent cell proliferation of a human breast epithelial line (HBL100), suggesting its potential role as a potent antiangiogenic substance. The derived cell line (HH9), which was transformed with the *hst/FGF4* gene, has been shown to be highly proliferative in vitro and to induce angiogenic tumours in nude mice. We show here that CMDB7 inhibits the mitogenic activities of the conditioned media from HBL 100 and HH9 cells in a dose-dependent manner. When HH9 cells were injected s.c. into nude mice, CMDB7 treatment (300 mg $kg^{-1}$ $week^{-1}$) suppressed the tumour take and the tumour growth by about 50% and 80% respectively. Immunohistochemical analysis showed a highly significant decrease, by more than threefold, in the endothelial density of viable tumour regions, together with a significant increase in the necrosis area. This antiangiogenic activity of CMDB7 was further demonstrated by direct inhibition of calf pulmonary artery (CPAE) and human umbilical vein (HUVEC) endothelial cell proliferation and migration in vitro. In addition, we showed that CMDB7 inhibits specifically the mitogenic effects of the growth factors that bind to heparin such as FGF-2, FGF-4, platelet-derived growth factor (PDGF-BB) and transforming growth factor (TGF-β1), but not those of epidermal growth factor (EGF) and insulin-like growth factor (IGF-1). These results demonstrate that CMDB7 inhibits FGF-2/FGF-4-dependent tumour growth and angiogenesis, most likely by disrupting the autocrine and paracrine effects of growth factors released from the tumour cells.

**Keywords:** dextran derivative; fibroblast growth factor; angiogenesis; breast cancer

Fibroblast growth factors (FGFs) are a nine-member family of proteins that exhibit potent mitogenic activity towards cells of mesenchymal, neuronal and epithelial origin (Basilico and Moscatelli, 1992; Tanaka et al, 1992; Miyamoto et al, 1993). FGFs require heparin as a co-factor for their interaction with the receptors. Although some FGFs do not possess a signal sequence, all of them appear to be secreted and accumulated in the extracellular matrix (Klagsbrun, 1989). In the human mammary gland, FGF-2 is localized in the area of myoepithelial and epithelial cells (Gomm et al, 1991). Clinical studies indicate that these growth factors may be involved in breast cancer development. For example, *FGF-1*, *FGF-2*, *FGF-receptor 1* (*FGF-R1*), *FGF-R2* and *FGF-R3* gene expression has been detected in breast cancer (Penault-Lorca et al, 1995), and *FGF3* and *FGF4* genes are up-regulated in 20% of breast cancer (Theillet et al, 1989). In some breast cancer patients FGF-2 is abnormally elevated in the serum or urine (Takei et al, 1993; Nguyen et al, 1994).

A number of studies have shown that FGFs are involved in tumorigenesis and metastasis in vivo (Dickson et al, 1984; Murakami et al, 1990; Wellstein et al, 1991). For example, MCF-7 cells transfected by FGF-4 become tumorigenic and metastatic when xenografted in nude mice, even in the absence of oestrogens (Mcleskey et al, 1993). FGF-2 exerts angiogenic activity in vivo (Czubayko et al, 1994) and induces proliferation, protease production and migration of endothelial cells in vitro (Sato and Rifkin, 1988; Tsuboi et al, 1990; Gualandris et al, 1994). FGF-2-overexpressing endothelial cells acquire an angiogenic phenotype and recruit quiescent endothelium originating in angioproliferative lesions in vivo (Gualandris et al, 1996).

In previous studies, we have shown that the FGF-2 autocrine pathway can be involved in tumour progression. The HBL100 cells, not tumorigenic in nude mice, produce and secrete FGF-2, which stimulates their growth in an autocrine manner (Souttou et al, 1994). The cell lines obtained by transfection of human breast epithelial HBL100 cells with the activated *FGF-4* oncogene develop colonies in soft agar and produce highly vascularized tumours as a result of the production and secretion of both FGF-2 and FGF-4, which can act as autocrine and paracrine factors. But the presence of FGF-1, insulin-like growth factors (IGF-1, IGF-2), transforming growth factors (TGF-α, TGFβ-1) and platelet-derived growth factor (PDGF-BB) is not detected in conditioned medium and in cell extracts of HH9 cells (Souttou et al, 1996). This cell line may provide an appropriate experimental model for studying antitumoral and anti-angiogenesis molecules that act on the autocrine and paracrine regulations of tumour development.

*Received 27 June 1997*
*Revised 12 November 1997*
*Accepted 16 December 1997*

*Correspondence to:* R Bagheri-Yarmand

In previous papers, we have shown that some dextran derivatives displayed an in vitro growth inhibitory activity on breast pretumour and tumour cells depending on their composition (Morère et al. 1992; Bagheri-Yarmand et al. 1994; Liu et al. 1997). For some of them, e.g. carboxymethyl benzylamide dextran (CMDB), a positive correlation was found between the inhibition of cell proliferation and the overall content of benzylamide. Growth inhibition was associated with a decrease in the proportion of S-phase cells and an accumulation of cells in $G_1$ phase (Bagheri-Yarmand et al. 1994). Recently, we showed that one member of these dextran derivatives, CMDB7, could exert its antiproliferative action on HBL100 cells by interfering with the FGF-2 autocrine growth of these cells (Bagheri-Yarmand et al. 1997). Experimental data that emphasize the growth inhibitory potential of this cytostatic non-toxic compound led us to examine the activity of CMDB7 on the breast cancer experimental model of HH9 tumour in nude mice. In this paper, using this experimental model, we investigated the effect of CMDB7 on: (a) the in vitro growth of HH9 cells; (b) the mitogenic activities of conditioned medium from HBL100 and HH9 cells on fibroblasts and endothelial cells; (c) the tumour take and tumour growth of HH9 cells in nude mice and the neovascularization of these tumours; (d) the in vitro migration and proliferation of endothelial cells and (e) the mitogenic activities of purified angiogenic growth factors on fibroblasts.

### MATERIALS AND METHODS

### Dextran derivative preparation

Water-soluble dextran derivative (CMDB7) was prepared from dextran T40 as previously described (Chaubet et al. 1995). In brief, CMDB7 was prepared by a statistical substitution of dextran in two steps: carboxymethylation, followed by coupling of benzylamide. This derivative was then purified by ultrafiltration and lyophilized. The chemical composition was determined by acidimetric titration and elementary analysis of nitrogen (dextran, 0%; carboxymethyl, 70%; benzylamide, 30%; mass apparent molecular weight = 80 000 g $mol^{-1}$).

### Cell lines

The breast epithelial cell line HBL100 obtained from Dr R Cassingena (Villejuif, France), was established from the milk of an apparently healthy woman harbouring SV40 genetic information (Polanowski et al. 1976). The ras transformed HBL100 cells (HBL100 ras) were obtained from Dr G Goubin (Institut Curie, France) (Lebeau et al. 1991). The HH9 cell line used in this study is a clone of HBL100 cells transformed with the *FGF-4* oncogene (Souttou et al. 1996). All epithelial cell lines and Balb/c 3T3 fibroblasts (ATCC) were grown in Dulbecco's modified Eagle's medium (DMEM) (Gibco, Life Technologies, France) supplemented with 2 mM L-glutamine, 50 IU $ml^{-1}$ penicillin and 50 µg $ml^{-1}$ streptomycin, and 10% fetal calf serum (FCS) (Gibco), at 37°C in a humidified atmosphere containing 5% $CO_2$.

Calf pulmonary artery endothelial CPAE cells (ATCC) were grown in MEM supplemented with 20% FCS, 2 mM L-glutamine, 50 IU $ml^{-1}$ penicillin and 50 µg $ml^{-1}$ streptomycin and used between passages 12 and 20. HUVEC cells were isolated from freshly delivered umbilical cords obtained from natural or caesarean births. These cells were grown in M199 supplemented with 20% FCS, 2 mM L-glutamine, 50 IU $ml^{-1}$ penicillin and 50 µg $ml^{-1}$ streptomycin and used between passages 1 and 2.

### Growth assays

Epithelial cells in their exponential growth phase were seeded at a density of 1.5 × $10^4$ cells per well in 24-well tissue culture plates (Polylabo, Strasbourg, France) for 24 h to allow cell attachment in medium with 10% FCS. They were then washed with DMEM and incubated in the medium with 1% FCS and various concentrations of CMDB7 (day zero). At different days, cells were harvested with 0.025% of trypsin–EDTA (Gibco) and the cell number of each well was determined in triplicate using a Coulter counter (Coultronics, Margency, France).

### Preparation of conditioned medium

Epithelial cells in 150-mm-diameter Petri dishes (Polylabo) were grown to 80% confluence in DMEM supplemented with 10% FCS, washed twice with DMEM and incubated in 10 ml per dish of DMEM containing 0.1% bovine serum albumin (BSA) (Sigma Chemical Co., France) for 24 h at 37°C. The medium was then harvested, cleared by centrifugation and stored at –80°C before use.

### Measurement of [³H]thymidine incorporation in DNA

Balb/c 3T3 fibroblasts ($10^5$ cells) were seeded in DMEM supplemented with 10% FCS into 24-well tissue culture plates, grown until confluence, washed twice with DMEM and incubated for 24 h in serum-free DMEM containing 0.1% BSA. This treatment rendered the cells quiescent. The medium was then replaced with conditioned medium (0.75 ml per well) and 0.25 ml of DMEM with or without CMDB7. Cells were incubated for an additional 20 h and then for 4 h with DMEM containing 1 µCi $ml^{-1}$ [³H]-thymidine (Amersham, Bucks, UK). After being washed with ice-cold PBS, the cells were fixed with ice-cold 10% trichloracetic acid, and solubilized by using 0.3 M NaOH. The radioactivity incorporated was counted using a Beckman scintillation counter. In experiments with purified growth factors, subconfluent cells were serum starved for 24 h and then incubated with recombinant human FGF-2 (Genzyme Diagnostics, Cambridge, MA, USA), recombinant human FGF-4 (R&D Systems, Abingdon, UK), ultrapure human TGF-β1, recombinant human PDGF-BB, recombinant human EGF, recombinant human IGF-1 (TEBU, France) for 20 h. Cells were then labelled for 4 h with 1 µCi $ml^{-1}$ [³H]thymidine. Trichloroacetic acid-precipitable radioactivity was then measured and expressed as c.p.m. incorporated per well.

### Endothelial cell proliferation assay

CPAE and HUVEC cells were cultured at 4000 cells per well tissue culture plated in 96 wells in complete medium containing 20% FCS. After 24 h, medium was removed and fresh medium with 5% FCS and various concentrations of CMDB7 were added to the cells. Twenty-four hours later, [³H]thymidine (Amersham, France) was added at 1 µCi per well. [³H]Thymidine incorporated following 18 h of culture was absorbed to a paper filter by a Skatron harvester (Skatron, Lier, Norway) and the radioactivity was determined in a liquid scintillation counter. Quadruplicate experiments were repeated at least three times.

### Migration assay of endothelial cells

A wound was created with a scraper in confluent endothelial monolayer in 35-mm tissue culture dishes (Falcon labware) so as

© Cancer Research Campaign 1998

to destroy half of the monolayer. Culture medium and detached cells were removed, the monolayer washed with culture medium and fresh complete medium added with or without tested agents. Transparent graph paper was then stuck to the bottom of the tissue culture dishes so that the shifting distance of the edge of migration could be directly measured. The results of the migration distance were expressed as a percentage of the migration rate of the control cells. The experiments were repeated at least three times.

### Xenografts in nude mice

Swiss nu/nu male athymic mice, 3 weeks old, were obtained from Charles River, France. Animals were kept in a temperature-controlled room on a 12:12 light–dark schedule with food and water *ad libitum*. HH9 cells were cultivated in DMEM supplemented with 10% FCS in T150 plates and harvested at 80% confluence. Cells ($10^7$) were inoculated s.c. into the right axillary region of the flank of nude mice. One week after the tumour cell inoculation, CMDB7 was injected s.c. close to tumours at a dose of 150 mg kg$^{-1}$ in 0.1 ml of phosphate-buffered saline (PBS), twice a week, for 9 weeks. The control group received s.c. a 0.1 ml injection of PBS. Tumours were measured along two major axes with calipers. Tumour volume was caculated as follows: $V = 0.5 \times R_1^2R_2$ where $R_1$is the shortest diameter and $R_2$ is the longest diameter. Animals (24 mice) were arbitrarily placed in control ($n$ = 12) and CMDB7 ($n$ = 12) groups.

### Tissue storage and immunohistochemical analysis

Tumour specimens were fixed with a solution of formalin (4%) immediately after surgical resection. The fixed samples were processed to paraffin in the usual way, and 5-μm sections were examined in haematoxylin and eosin preparations. Immunohistochemical staining using a Universal Kit of LSAB2 (K675-DAB; Dako, France) was also performed using monoclonal mouse antibodies against Ki-67 (MIB-1; Immunotech). The number of positive cells for Ki-67 monoclonal antibody was estimated in six high-power fields containing 60–80 cells per field (× 400). Endothelial cells were specifically stained with Griffonia (Banderaea) simplicifolia lectin (GSL-1). The GSL-1 lectin binds specifically to a-galactosyl residues and marks the vascular endothelium in mice (Alroy et al, 1987). Sections (5 μm) were deparaffinized and rehydrated. The endogenous peroxidase was inactivated with 3% $H_2O_2$ and washed in Tris-buffered saline (TBS), pH 7.6, followed by preincubation in FCS for 30 min at room temperature. The sections were then incubated for 45 min with biotinylated GSL-1 (Vector Laboratories, Burlingame, CA, USA) at a concentration of 0.01 mg ml$^{-1}$, washed with TBS treated for 30 min with an avidin–biotin–peroxidase complex (Vector laboratories) and washed again with TBS. The peroxidase was activated by incubation for 10 min in 0.1 M acetate buffer (pH 5.2) containing 3% $H_2O_2$ and 3% 3-amino-9-ethylcarbazole. Finally, the slides were washed in distilled water and tap water, counterstained with haematoxylin, dehydrated and coverslipped with permount. The analysis was performed on regions containing exclusively viable tumour cells depicted by the haematoxylin stain. In each GSL-1-stained section of control and of CMDB7-treated tumours, five areas in exclusively viable regions were selected randomly. Image analysis of the sections stained for endothelial cells with GSL-1 was performed on a Macintosh (Power Macintosh 8500/120) computer using the public domain NIH image program (developed at the US National Institutes of Health and available on the Internet at http://rsb.info.nih.gov/nih-image/). The area of stained endothelial cells was determined with an experimental error of less than 5% by measuring the area above a threshold intensity of stain in each slice. The ratio of stained area to viewed area yielded an average endothelial density. The densities were then averaged in control tumours and in tumours treated with CMDB7.

**Figure 1** Inhibition of HH9 cell proliferation by CMDB7. HH9 cells were plated at a density of 1.5 × $10^4$ cells per well in 24-well plates in DMEM containing 10% FCS. On the following day (day 0), the medium was changed to DMEM with 1% FCS (■) containing 1 μM (□), 5 μM (○), 10 μM (●), 25 μM (△) and 50 μM (▲) of CMDB7. At the indicated days, cells were trypsinized and counted by using a Coulter counter. Values provided from one of the three independant experiments performed in triplicate (mean ± s.d.)

### Statistical analysis

In the figures, the data are presented as the mean values for a 95% confidence interval. Multiple statistical comparisons were performed using ANOVA and Mann–Whitney in a multivariate linear model.

### RESULTS

### Inhibition of the HH9 cell growth by CMDB7

Increasing concentrations of CMDB7 ranging from 1 μM to 50 μM added to HH9 cells resulted in a dose-dependent inhibition of cell number (Figure 1). We observed a cytostatic effect of CMDB7 with an $IC_{50}$ of about 25 μM after 8 days of treatment (Figure 1). When CMDB7 was removed from the culture medium after a 4-day treatment, cell growth resumed at a rate similar to that observed in untreated cells (data not shown). HH9 cells cultured with CMDB7 appeared otherwise healthy; they remained attached to the plastic tissue culture flask and did not undergo any noticeable morphological changes. In order to confirm the specific effects of CMDB7 on FGF-2 and FGF-4, we tested the effect of CMDB7 on ras transformed HBL100-cells. As mutated ras functions downstream of FGF receptor stimulation, CMDB7 did not affect the growth of these cells (data not shown).

© Cancer Research Campaign 1998

### Inhibitory effect of CMDB7 on the paracrine activity of conditioned medium from HBL100 and HH9 cells on fibroblasts and endothelial cells

We previously showed that HH9 cells secrete large amounts of FGF-2 and FGF-4 in the conditioned medium (CM: Souttou et al. 1996). Conditioned media derived from parental HBL100 (CM-HBL100) and HH9 (CM-HH9) cells were tested for their ability to stimulate [$^3$H]thymidine incorporation into the DNA of Balb/c3T3 cells in the presence or absence of CMDB7. CM obtained from HBL100 and HH9 cells stimulated 2.4- and 3.4-fold respectively the [$^3$H]thymidine DNA uptake in fibroblasts. These stimulations were dose-dependently inhibited by CMDB7: 99% inhibition was found in a concentration of 5 μM for CM-HBL100 and 63% inhibition at 50 μM for CM-HH9 (Figure 2A). CMDB7 alone did not affect the proliferation of fibroblasts in serum-free medium. We determined that media conditioned by HBL100 and HH9 cells stimulated the growth of endothelial cells (CPAE) about 15- and 22-fold above control respectively. On CPAE cells, CMDB7 (50 μM) inhibited the paracrine effect of growth factor(s) secreted by HBL100 and HH9 cells by about 70% and 57% respectively (Figure 2B).

### Effect of CMDB7 on HH9 cells xenografted in nude mice

To study the CMDB7 effect on the tumour appearance, one week after s.c. inoculation of $10^7$ exponentially growing HH9 cells in the right flank of mice, we injected at the same site, twice per week, 4 mg of CMDB7 per mouse (150 mg $kg^{-1}$). Palpable tumours appeared in 60%, 70% and 90% of control mice 3, 4 and 8 weeks after cell injections respectively. In mice treated with CMDB7, palpable tumours appeared in 15%, 40% and 50% of mice 5, 6 and 10 weeks after cell injection respectively (Figure 3A). CMDB7 decreased the tumour size by 80% after 9 weeks of treatment in the case of treated mice that developed a tumour ($P < 0.00001$) (Figure 3B). By measuring proliferating tumour cells with antibody against nuclear Ki-67 antigen, we showed that all the proliferating activities were similar, both in untreated and treated tumours (Table 1). Immunostaining with anti-FGF-2 of HH9 tumours showed that FGF-2 is present in these cells (data not shown). Necrotic areas in treated tumours (53.4 ± 4.1) were increased compared with untreated tumours (34.2 ± 5.2) ($P < 0.001$) (Table 1). To determine whether the CMDB7-enhanced necrosis was directly associated with a quantitative reduction in tumour vascularity, we measured the density of the endothelial cells that composed the microcapillaries in the regions of viable tumour cells close to the tumour periphery. A specific biotinylated lectin interacting with endothelial cells (GSL-1) enabled us to stain these cells selectively (Figure 4). The density of the endothelial cells (percentage of area occupied by endothelials cells) was determined by image analysis of the stained area. The mean percentage of endothelial cell area of control tumours was 7.5 ± 1.5 and of CMDB7-treated tumours was 2.39 ± 0.6 (Table 1). The reduced endothelial cell density as a result of CMDB7 treatment was highly significant ($P < 0.0001$).

### CMDB7 inhibits the in vitro endothelial cell proliferation

To test the possibility that CMDB7 exerts its antiangiogenic activity by impairing endothelial cell proliferation, the effect of CMDB7 on the CPAE and HUVEC cell growth was determined in vitro. CMDB7 inhibited these two endothelial cell proliferations in a dose-dependent manner. The calculated $IC_{50}$ of CMDB7 inhibition on CPAE and HUVEC cell proliferation was 8 μM and 4 μM respectively (Figure 5A).

**Figure 2** Effect of conditioned medium (CM) from HBL100 or HH9 cells on the DNA synthesis of Balb/c3T3 fibroblasts and endothelial cells in the absence or presence of CMDB7. Subconfluent cultures of Balb/c 3T3 fibroblasts (A) or endothelial cells (CPAE) (B) were growth arrested by serum starvation for 24 h and then incubated for 20 h in the presence of HBL100- or HH9-conditioned medium in the presence or absence of various concentrations of CMDB7. [$^3$H]Thymidine incorporation was performed for 4 h, and trichloroacetic acid-precipitable radioactivity was counted. Values provided from one of the three independent experiments performed in triplicate (mean ± s.d.)

### CMDB7 inhibits the in vitro endothelial cell migration

Endothelial cell migration is one of the critical features of neovascularization and wound repair. Therefore, we examined the effect of CMDB7 on the in vitro movement of CPAE and HUVEC cells from an in vitro wound edge. The wound was made by removing a patch of cells from a confluent monolayer with a razor blade. Endothelial cell migration was determined by the distance travelled by the cells through the wound edge. The addition of

© Cancer Research Campaign 1998

**Figure 3** Effect of CMDB7 on tumour take and tumour growth in nude mice. HH9 cells ($10^7$) were inoculated s.c. into the right axillary region of the flank of male nude mice, day 0 ($n$ = 24). Treatment was started 1 week after subcutaneous inoculation of tumour cells and stopped after 9 weeks. CMDB7 was administered s.c. twice a week at 150 mg $kg^{-1}$ (○) ($n$ = 12). Control mice were injected with PBS (●) ($n$ = 12). **A** Values represent percentage of tumour/number of mice. **B** Tumour volumes ($mm^3$) are the mean ± s.e.m. of mice bearing a tumour

CMDB7 into the medium without serum inhibited the migration of the cells into the denuded area (Figure 5B). The CMDB7 inhibition of cell movement occurred in a dose-dependent manner. Thus, 49% and 31% inhibitions of CPAE and HUVEC cell movement were obtained respectively at a CMDB7 concentration of 20 μM after 24 h (Figure 5B).

**Table 1** Changes in percentage of necrosis and in percentage of endothelial cell density in control and CMDB7-treated tumours but not in proliferation index[a]

| | Control tumours ($n$ = 11) | CMDB7 tumours ($n$ = 6) |
|---|---|---|
| Index Ki-67 | 80 ± 2[b] | 78 ± 1 |
| Necrosis (%) | 34.2 ± 5.2 | 53.4 ± 4.1[c] ($P$ < 0.001) |
| Area of endothelial cells (%) | 7.5 ± 1.5 | 2.39 ± 0.6[c] ($P$ < 0.0001) |

[a]Mean ± s.d. [b]The number of positive cells for Ki-67 monoclonal antibody was estimated in six high-power fields containing 60–80 cells per field at × 400 magnification. Results were described as percentage of cells stained. [c]A statistically significant change relative to control.

**Figure 4** Microscopic view of histological sections of endothelial cells stained with GSL-1. **A** Control tumours. **B** Treated tumours by CMDB7. The details of the staining procedure are given in Materials and methods (original magnification × 400)

### Inhibitory effect of CMDB7 on fibroblast stimulation by FGF-2, FGF-4, PDGF-BB and TGF-β1 but not EGF and IGF-1

We studied the mitogenic potency of FGF-2, FGF-4, PDGF-BB, TGF-β, EGF and IGF-1 in the presence or absence of CMDB7 on Balb/c3T3 fibroblasts. Figure 6 shows that the addition of 2 ng $ml^{-1}$ FGF-2 or FGF-4 enhanced the cell growth about nine- and sixfold respectively. Under these experimental conditions, CMDB7 (50 μM) inhibited the FGF-2 cell growth stimulation by

**Figure 5** Effect of CMDB7 on proliferation (**A**) and migration (**B**) of CPAE and HUVEC. **A** After culture of CPAE and HUVEC in the media containing 5% FCS for 24 h and futher culture in the media containing various concentrations of CMDB7 for 24 h, [$^3$H]thymidine was added for 18 h. **B** Confluent endothelial cells were wounded with a razor blade. The cells were incubated for 24 h with various concentrations of CMDB7. Evaluation of the migration distance was expressed as a percentage of the migration rate of the control cells. The experiments were repeated at least three times

**Figure 6** Effect of various growth factors on the DNA synthesis of Balb/c 3T3 in the presence or absence of CMDB7. Subconfluent cultures of Balb/c 3T3 fibroblasts were growth arrested by serum starvation for 24 h and then incubated for 20 h in the presence of recombinant FGF-2, FGF-4, TGF-β, EGF, IGF-1 (2 ng ml$^{-1}$) and PDGF-BB (10 ng ml$^{-1}$) in the presence or absence of CMDB7 (50 μM). [$^3$H]Thymidine incorporation was performed for 4 h, and trichloroacetic acid-precipitable radioactivity was counted. Values provided from one of the three independent experiments performed in triplicate (mean ± s.d.)

55% and the FGF-4 cell growth stimulation by 30% ($P < 0.01$) (Figure 6). In Balb/c3T3 fibroblasts, [$^3$H]thymidine DNA incorporation was stimulated to about 5.5-fold when cells were exposed to PDGF-BB (10 ng ml$^{-1}$) for 24 h. CMDB7 (10 μM) reduced the stimulation of fibroblast DNA synthesis by PDGF-BB to only 2.9-fold ($P < 0.0001$) (Figure 6). Incubation of fibroblasts with TGF-β1 (2 ng ml$^{-1}$) increased the [$^3$H]thymidine incorporation into fibroblast DNA by about 7.6-fold. CMDB7 (10 μM), for 24 h, completely abolished the TGF-β1-induced DNA synthesis ($P < 0.0002$). CMDB7 alone induced no changes in the level of Balb/c3T3 DNA synthesis compared with the control (Figure 6). Under the same conditions, EGF and IGF-1 enhanced the thymidine uptake by about three- and twofold respectively. So CMDB7 did not effect EGF and IGF-1 growth stimulation (Figure 6).

### DISCUSSION

Previous studies have shown that CMDB7 inhibits HBL100 cell growth by interfering with the FGF-2 autocrine loop in these cells (Bagheri-Yarmand et al, 1997). In this paper, we have shown that the in vitro proliferation of HH9 cells obtained by transformation of HBL100 cells with the *hst/FGF4* gene are also inhibited in the presence of CMDB7. The CMDB7 treatment of HH9 tumour cells delayed the appearance of tumours and reduced tumour incidence. Only 50% of treated mice developed a tumour after 10 weeks vs 90% in control mice. In addition, treated tumour volume was only 20% of that in the control group at the end of the treatment. The number of proliferating Ki-67-positive tumour cells is identical in control and treated tumours. Furthermore, in the CMDB7-treated tumours a reduction of 68% in the number of capillary vessels has occurred in viable tumour regions, indicating that this treatment was attenuating the rate of neovascularization but did not completely block the initial activation of angiogenesis, nor the capability of every capillary to grow. In addition, CMDB7-treated tumours have more plurifocal necrosis. This reduction in endothelial cell density and concomitant increase in necrosis lends support to our hypothesis that CMDB7, by inhibiting angiogenesis and endothelium growth, impairs delivery of nutrients and oxygen, thereby leading to cell death. These results are in agreement with a number of studies that demonstrated that angiogenesis plays an essential role in tumour growth (Folkman, 1990; Kim et al, 1993; Vartanian and Weidner, 1994). Inhibition of angiogenesis by means of FGF-2 immunoneutralizing monoclonal antibody has been reported to be associated with anti-tumour effect in vivo (Hori et al, 1991), suggesting that novel therapeutic approaches to cancer therapy might involve the use of antiangiogenic drugs.

© Cancer Research Campaign 1998

In order to explain this CMDB7 antiangiogenic effect, we have tested the CMDB7 effect on endothelial cell proliferation, a key step in the angiogenesis process (Folkman and Klagsbrun, 1987). CPAE and HUVEC cell proliferation was inhibited by CMDB7. It is unknown whether this inhibition was due to FGF-2-independent mechanisms or the inhibition of an autocrine FGF-2 production by endothelial cells (Schweigerer et al, 1987). Moreover, this inhibition of endothelial cell proliferation could be due to interference with the binding of angiogenic factors such as FGF-2 or FGF-4 to endothelial cells (Vaisman et al, 1990). In accord with this hypothesis, we have shown that CMDB7 binds to FGF-2 and prevents the binding of radiolabelled FGF-2 to its high- and low-affinity receptors on HBL100 cells (Bagheri-Yarmand et al, 1997). Migration of endothelial cells, which is also a key step in the angiogenesis process, mediated by FGF-2 (Sato and Rifkin, 1988), appears less sensitive to CMDB7 than does endothelial cell proliferation. Thus, CMDB7 can block neovascularization by directly inhibiting the angiogenic activity of endothelial cells.

Furthermore, the reduction in endothelium density caused by CMDB7 could result from its anti-growth factor activity, blocking paracrine stimulations of FGF-2 and FGF-4 on both fibroblasts and endothelial cells. Growth factors secreted by HBL100 and HH9 cells stimulated the incorporation of [$^3$H]thymidine by quiescent cultures of Balb/c3T3 fibroblasts. As FGF-1, IGF-1, IGF-2, TGF-$\alpha$, TGF-$\beta$1 and PDGF-BB are not detected in the conditioned media of HH9 cells, stimulation of the fibroblasts' DNA synthesis by HH9-conditioned medium is essentially due to FGF-2 and FGF-4. The CMDB7 treatment inhibited dose dependently CM-induced mitogenicity in Balb/c3T3 fibroblasts. However, CMDB7 at 5 $\mu$M completely blocks the mitogenic effect of factors secreted by HBL100 cells but inhibits partially (63%), at 50 $\mu$M, the stimulation induced by CM from HH9 cells. A possible explanation is an insufficient dose of CMDB7 for neutralizing the higher quantity of growth factors secreted in CM from HH9 cells compared with HBL100 cells (Souttou et al, 1996). The mitogenic effects of purified FGF-2, FGF-4, PDGF-BB and TGF-$\beta$1 on Balb/c3T3 fibroblast DNA synthesis is inhibited by CMDB7. In contrast, CMDB7 did not affect DNA stimulation by EGF and IGF-1.

CM from HBL100 and HH9 cells stimulated the growth of endothelial cells, 15- and 22-fold above the control respectively vs 2.4- and 3.4-fold stimulation on fibroblasts, suggesting that endothelial cells are more sensitive to FGF-2 and FGF-4 factors than fibroblasts. As CMDB7 partially inhibits the paracrine stimulation of conditioned media from HBL100 and HH9 cells on endothelial cells, it is likely that there is not a sufficient amount of CMDB7 to complex completely these angiogenic factors.

It was proposed that tumours might release factors able to stimulate expression, production and release of FGF-2 in and from capillary endothelial cells and so stimulate angiogenesis. Once the tumours are invaded by the capillaries, local release of FGF-2 could further enhance the growth of FGF-sensitive tumours (Schweigerer et al, 1987). We believe that the in vivo effect of CMDB7 is most probably due to its interaction with the FGF-2 and FGF-4 released from tumours. Mammary stromal fibroblasts may also produce factors that exert influence on the growth and malignant progression of breast tumours via paracrine effects on tumour-associated endothelium (Hlatky et al, 1994).

In conclusion, the present report shows that CMDB7 is an effective inhibitor of FGF-2 and FGF-4 mitogenic activity for fibroblasts and endothelial cells in vitro and a potent inhibitor for angiogenesis-dependent tumour growth in vivo. Thus, this compound should be an interesting candidate for developing new anti-cancer drugs, not only because of its potent inhibitory effect on tumour induced angiogenesis, but also because of its extremely low toxicity in vitro as well as in vivo.

### ACKNOWLEDGEMENTS

We thank Dr Y Legrand for discussions and review of the manuscript. We also thank Dr AM Rath and Dr R Vassy for discussions and technical advice. This work was supported by 'Banque de la vie, Recherche Cancer'.

### REFERENCES

Alroy J, Goyal V and Skutelsky E (1987) Lectin histochemistry of mammalian endothelium. *Histochemistry* **86**: 603–607

Bagheri-Yarmand R, Bittoun P, Champion J, Letourneur D, Jozefonvicz J, Fermandjian S and Crépin M (1994) Carboxymethyl benzylamide dextrans inhibit breast cell growth. *In vitro Cell Dev Biol* **30A**: 822–824

Bagheri-Yarmand R, Liu JF, Ledoux D, Morère JF and Crépin M (1997) Inhibition of human breast epithelial HBL100 cell proliferation by a dextran derivative (CMDB7): interference with the FGF2 autocrine loop. *Biochem Biophys Res Comm* **239**: 424–428

Basilico C and Moscatelli D (1992) The FGF family of growth factors and oncogenes. *Cancer Res* **59**: 115–164

Chaubet F, Champion J, Maïga R, Maurey S and Jozefonvicz J (1995) Synthesis and structure-anticoagulant property relationships of functionalized dextrans: CMDBS. *Carbohydrate Polymers* **28**: 145–152

Czubayko F, Smith RV, Chung HC and Wellstein A (1994) Tumour growth and angiogenesis induced by a secreted binding protein for fibroblast growth factors. *J Biol Chem* **269**: 28243–28248

Dickson C, Smith R, Brookes S, Peters G (1984) Tumorigenesis by mouse mammary tumour virus: proviral activation of a cellular gene in the common integration region int-2. *Cell* **37**: 529–536

Folkman J (1990) What is the evidence that tumours are angiogenesis dependent? *J Natl Cancer Inst* **82**: 4–6

Folkman J and Klagsbrun M (1987) Angiogenic factors. *Science* **235**: 442–447

Gomm JJ, Smith J, Ryall GK, Baillie R, Turnbull L and Coombes RC (1991) Localisation of basic fibroblast growth factor and transforming growth factor $\beta$1 in the human mammary gland. *Cancer Res* **51**: 4685–4692

Gualandris A, Urbinati C, Rusnati M, Ziche M and Presta M (1994) Interaction of high molecular weight basic fibroblast growth factor (bFGF) with endothelium: biological activity and intracellular fate of human recombinant mr 24000 bFGF. *J Cell Physiol* **161**: 149–159

Gualandris A, Rusnati M, Belleri M, Nelli EE, Bastaki M, Molinari-Tosatti MP, Bonardi F, Parolini S, Albini A, Morbidelli L, Ziche M, Corallini A, Possati L, Vacca A, Ribatti D and Presta M (1996) Basic fibroblast growth factor overexpression in endothelial cells: an autocrine mechanism for angiogenesis and angioproliferative diseases. *Cell Growth Differ* **7**: 147–160

Hlatky L, Tsionou C, Hahnfeldt P, Coleman CN (1994) Mammary fibroblasts may influence breast tumor angiogenesis via hypoxia-induced vascular endothelial growth factor up-regulation and protein expression. *Cancer Res* **54**: 6083–6086

Hori A, Sasada R, Matsutani E, Naito K, Sakura Y, Fujita T and Kazai Y (1991) Suppression of solid tumour growth by immunoneutralizing monoclonal antibody against human basic fibroblast growth factor. *Cancer Res* **51**: 6180–6184

Kim KJ, Bing L, Winer J, Armanini M, Gillett N, Phillips HS and Ferrara N (1993) Inhibition of vascular endothelial growth factor-induced angiogenesis suppresses tumour growth in vivo. *Nature* **362**: 841–845

Klagsbrun M (1989) The fibroblast growth factor family: structural and biological properties. *Prog Growth Factor Res* **1**: 207–235

Lebeau J, Chalony L, Prosperi MT and Goubin G (1991) Constitutive overexpression of a 89 kDa heat shock protein gene in the HBL100 human mammary cell line converted to a tumorigenic phenotype by the EJ/T24 Harvey-ras oncogene. *Oncogene* **6**: 1125–1132

Liu JF, Bagheri-Yamand R, Xia Y and Crépin M (1997) Modulations of breast fibroblast and carcinoma cell interactions by a dextran derivative (CMDB7). *Anticancer Res* **17**: 253–258

Mcleskey SW, Kurebayashi J, Honig SF, Zwiebel J, Lippman ME, Dickson RB and Kern FG (1993) Fibroblast growth factor 4 transfection of MCF-7 cells produces cell lines that are tumourigenic and metastatic in ovariectomized of tamoxifen-treated athymic nude mice. *Cancer Res* **53**: 2168–2177

Miyamoto M, Naruo K, Seko C, Matsumoto S, Kondo T and Kurokawa T (1993) Molecular cloning of a novel cytokine cDNA encoding the ninth member of the fibroblast growth factor family, which has a unique secretion property. *Mol Cell Biol* **13**: 4251–4259

Morère JF, Planchon P, Letourneur D, Avramoglou T, Jozefonvicz J, Israel L and Crépin M (1992) Inhibitory effect of substituted dextrans on MCF7 human breast cancer cell growth in vitro. *Anticancer Drug* **3**: 629–634

Murakami A, Tanaka H and Matsuzawa A (1990) Association of hst gene expression with metastatic phenotype in mouse mammary tumours. *Cell Growth Differ* **1**: 1–5

Nguyen H, Watanabe H, Budson AE, Richie JP, Hayes DF and Folkman J (1994) Elevated levels of an angiogenic peptide, basic fibroblast growth factor, in the urine of patients with a wide spectrum of cancers. *J Natl Cancer Inst* **86**: 356–361

Penault-Lorca F, Bertucci F, Adelaide J, Parc P, Coulier F, Jacquemier J, Birnbaum D and Delapeyriere O (1995) Expression of FGF and FGF receptor genes in human breast cancer. *Int J Cancer* **61**: 170–176

Polanowski F, Gaffney EV and Burke RE (1976) HBL-100, a cell line established from human milk. *In vitro Cell Dev Biol* **12**: 328–328

Sato Y and Rifkin DB (1988) Autocrine activities of basic fibroblast growth factor: regulation of endothelial cell movement, plasminogen activator synthesis, and DNA synthesis. *J Cell Biol* **110**: 1199–1205

Schweigerer L, Neufeld G, Friedman J, Abraham JA, Fiddes JC and Gospodarowicz D (1987) Capillary endothelial express basic fibroblast growth factor, a mitogen that promotes their own growth. *Nature* **325**: 257–259

Souttou B, Hamelin R and Crépin M (1994) FGF2 as an autocrine growth factor for immortal human breast epithelial cells. *Cell Growth Differ* **5**: 615–623

Souttou B, Gamby C, Crépin M and Hamelin R (1996) Tumoral progression of human breast epithelial cells secreting FGF2 and FGF4. *Int J Cancer* **68**: 675–681

Takei Y, Kurobe M, Uchida A and Hayashi K (1993) Presence of high-molecular-weight basic growth-factor-like immunoreactive substance in sera of patients with breast cancer. *J Clin Biochem Nutr* **15**: 57–64

Tanaka A, Miyamoto K, Minamino N, Takeda M, Sato B, Matsuo H and Matsumoto K (1992) Cloning and characterization of an androgen-induced growth factor essential for the androgen-dependent growth of mouse mammary carcinoma cell. *Proc Natl Acad Sci* **89**: 8928–8932

Theillet C, Le Roy X, Delapeyriere O, Grosgeorges J, Adnane J, Raynaud SD, Simony-Lafontaine J, Goldfarb M, Escot C and Birnbaum D (1989) Amplification of FGF-related genes in human tumors: possible involvement of HST in breast carcinomas. *Oncogene* **4**: 915–922

Tsuboi R, Sato Y and Rifkin DB (1990) Correlation of cell migration, cell invasion, receptor number, proteinase production, and basic fibroblast growth factor levels in endothelial cells. *J Cell Biol* **110**: 511–517

Vaisman N, Gospodarowicz D and Neufeld G (1990). Characterization of the receptor for vascular endothelial growth factor. *J Biol Chem* **256**: 19461–19466

Vartanian RK and Weidner N (1994) Correlation of intratumoural endothelial cell proliferation with microvessel density (tumour angiogenesis) and tumour cell proliferation in breast carcinoma. *Am J Pathol* **6** (144): 1188–1194

Wellstein A, Zugmaier G, Califano JA, Kern F, Paik S and Lippman ME (1991) Tumor growth dependent on Kaposi's sarcoma-derived fibroblast growth factor inhibited by pentosan polysulfate. *J Natl Cancer Inst* **83**: 716–720

© Cancer Research Campaign 1998


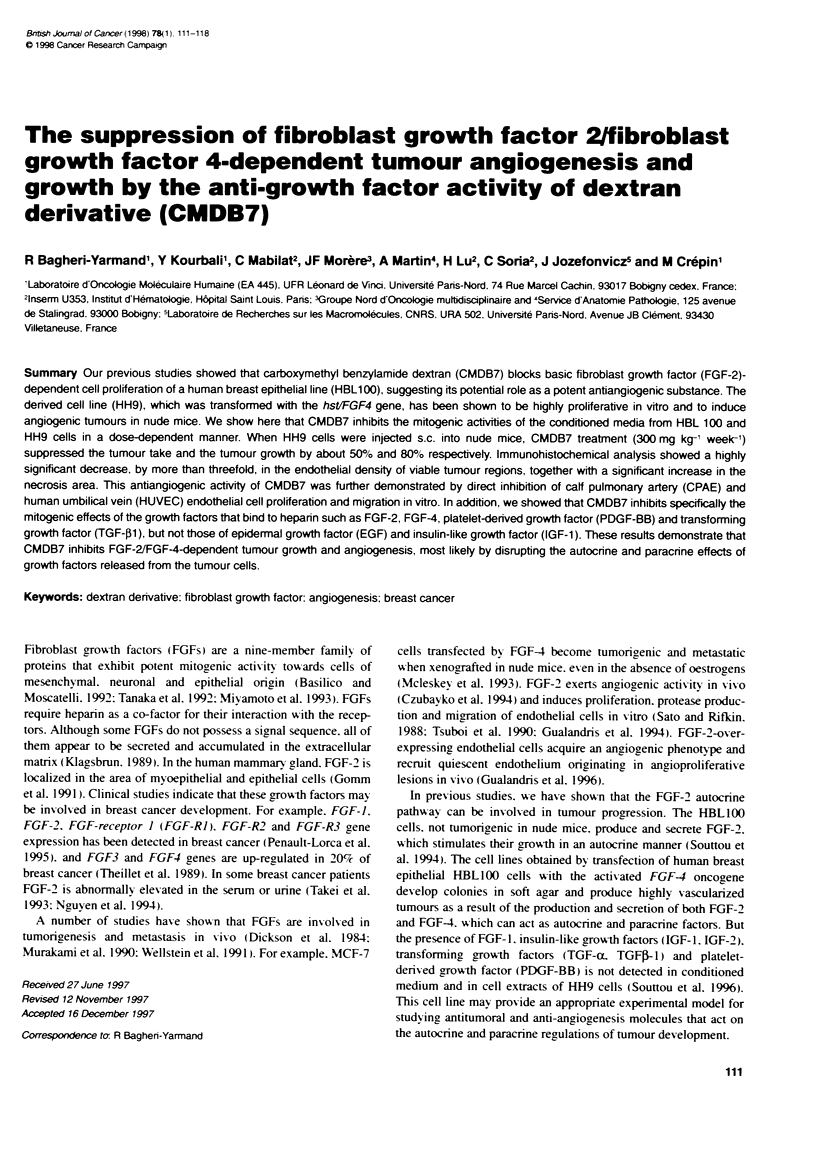

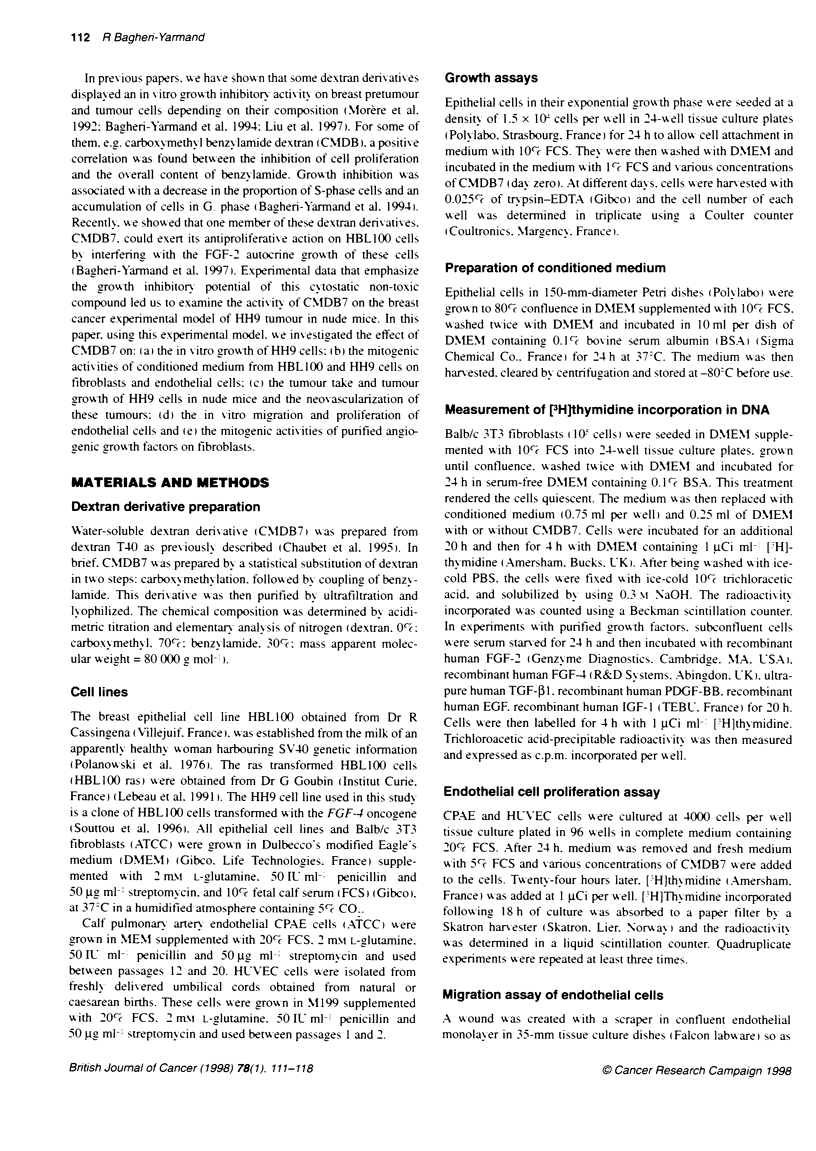

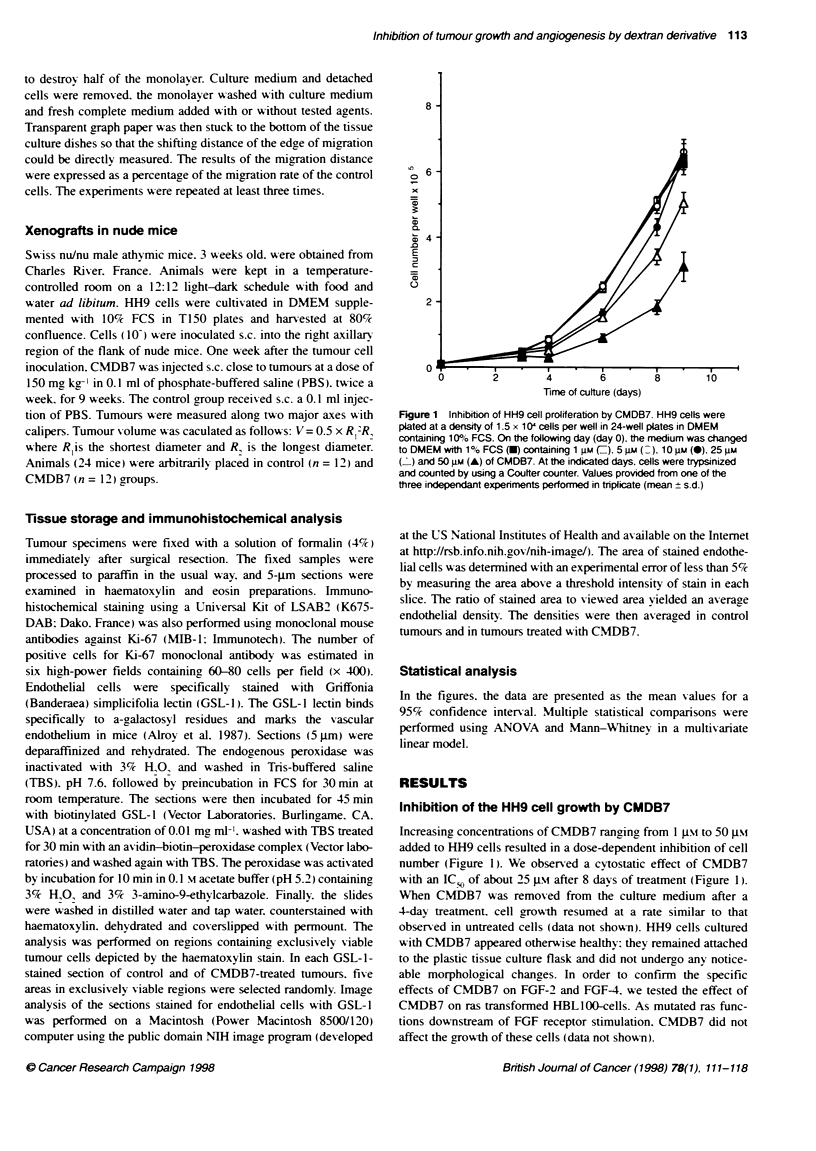

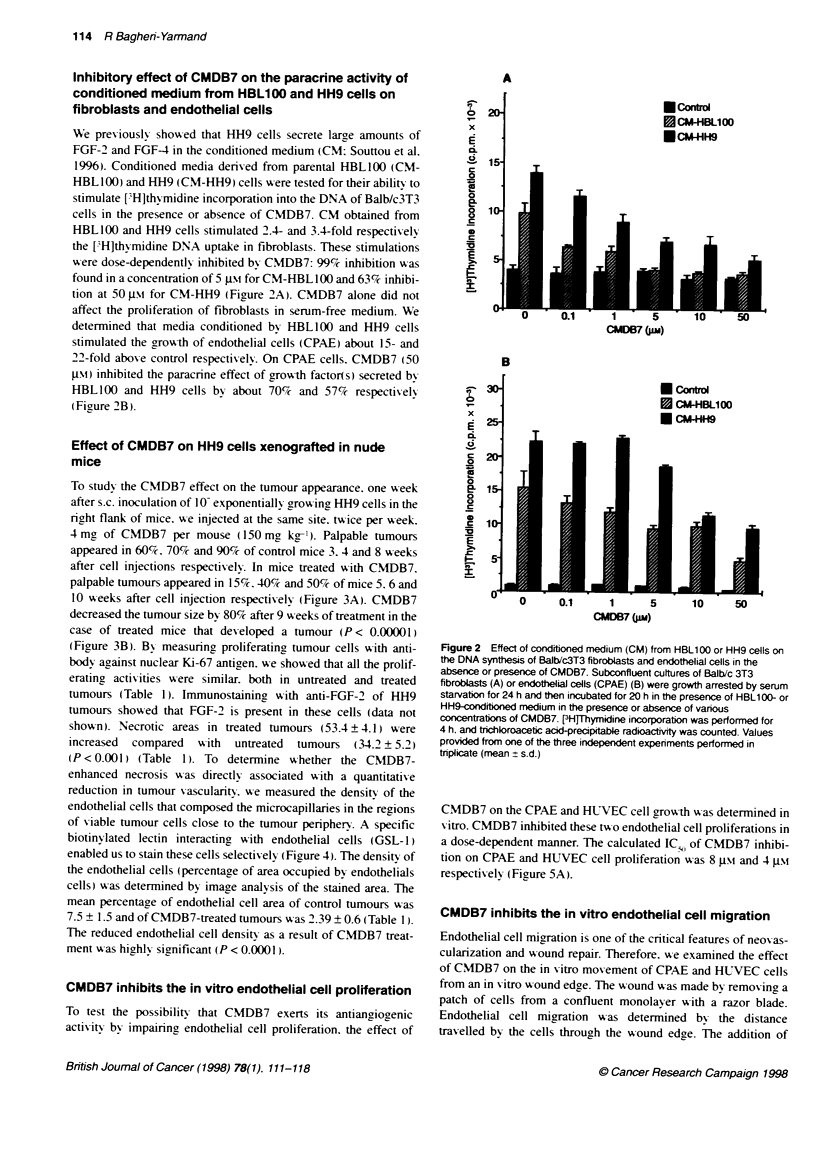

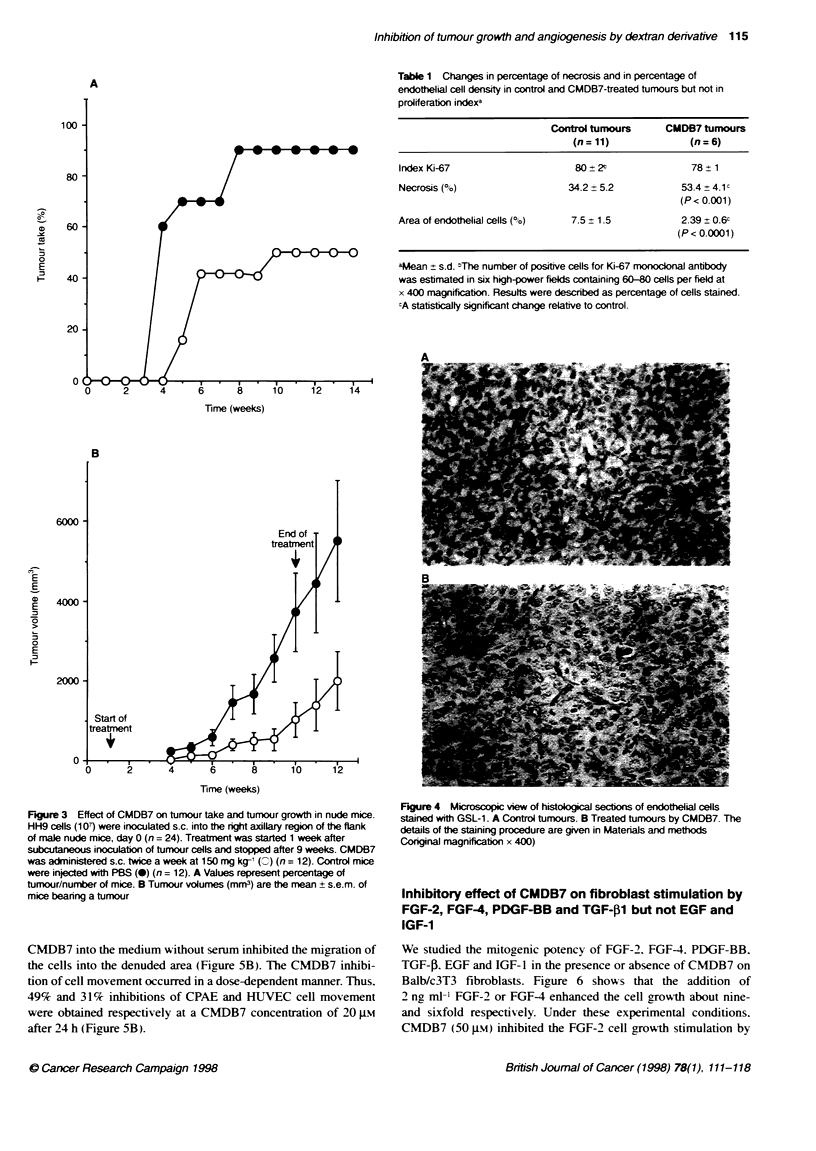

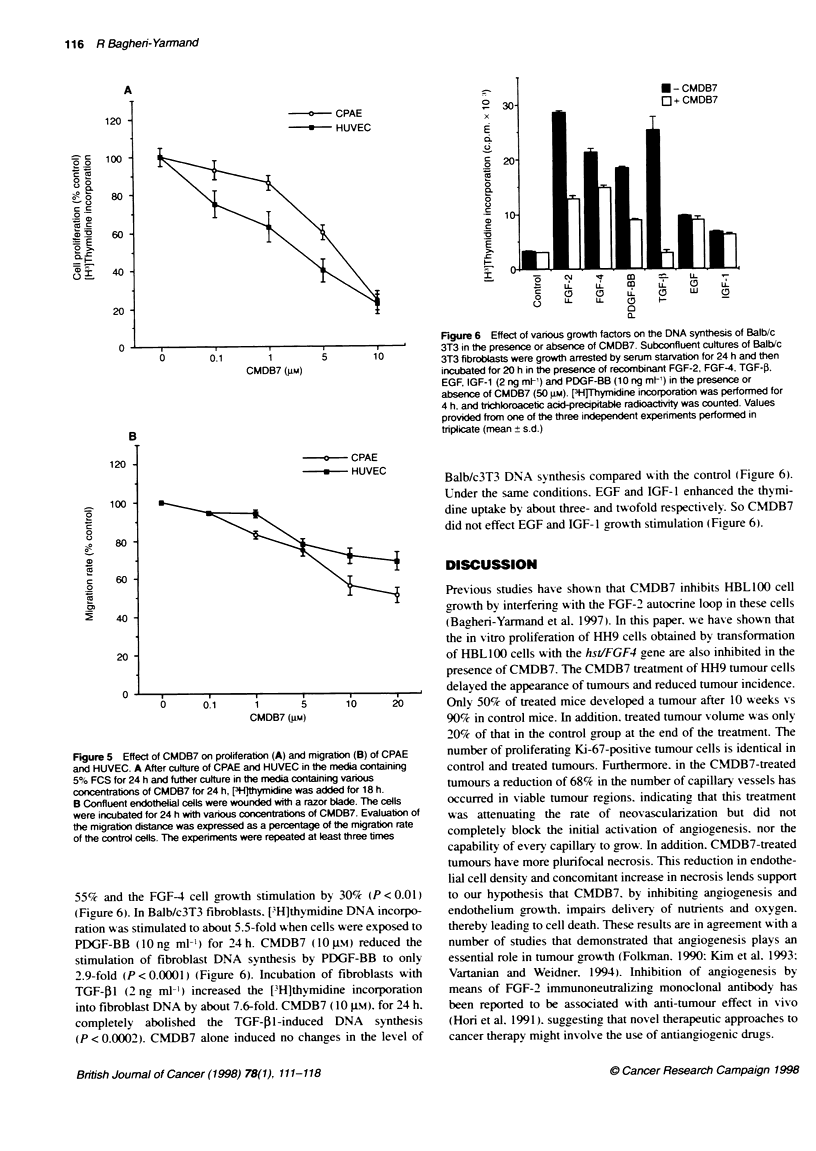

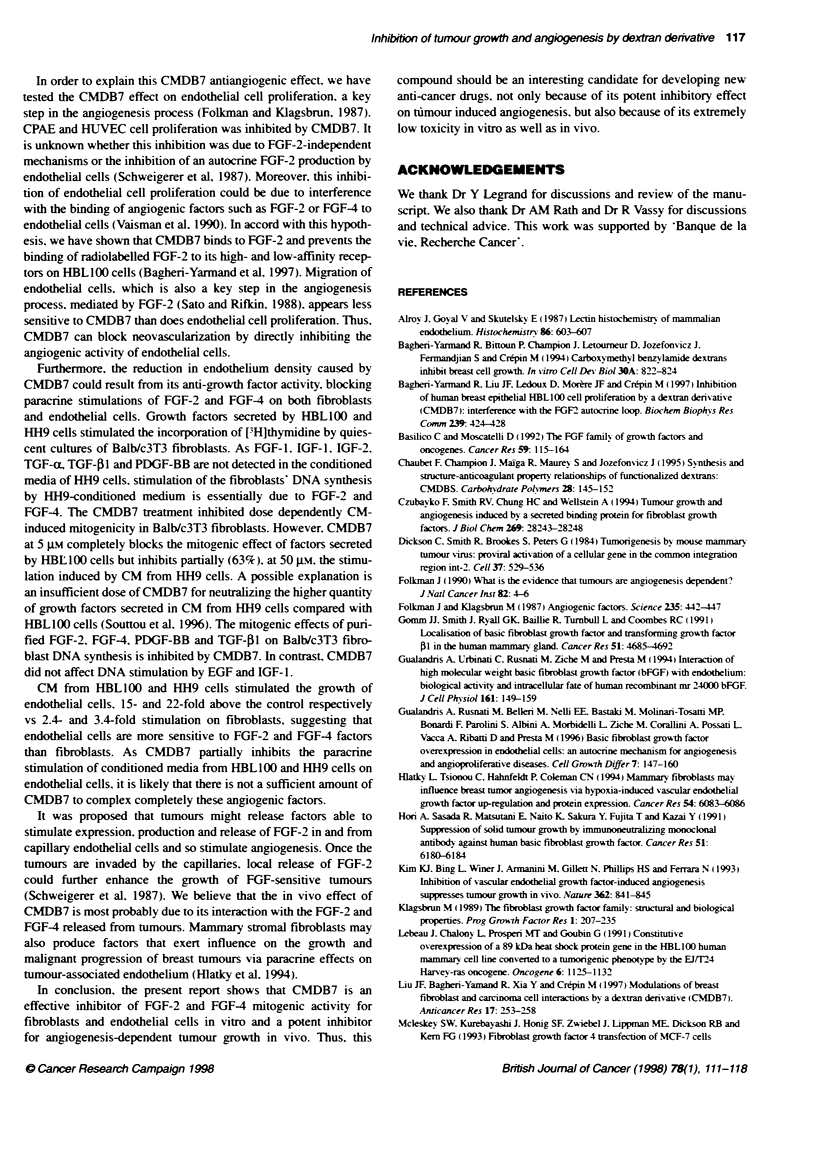

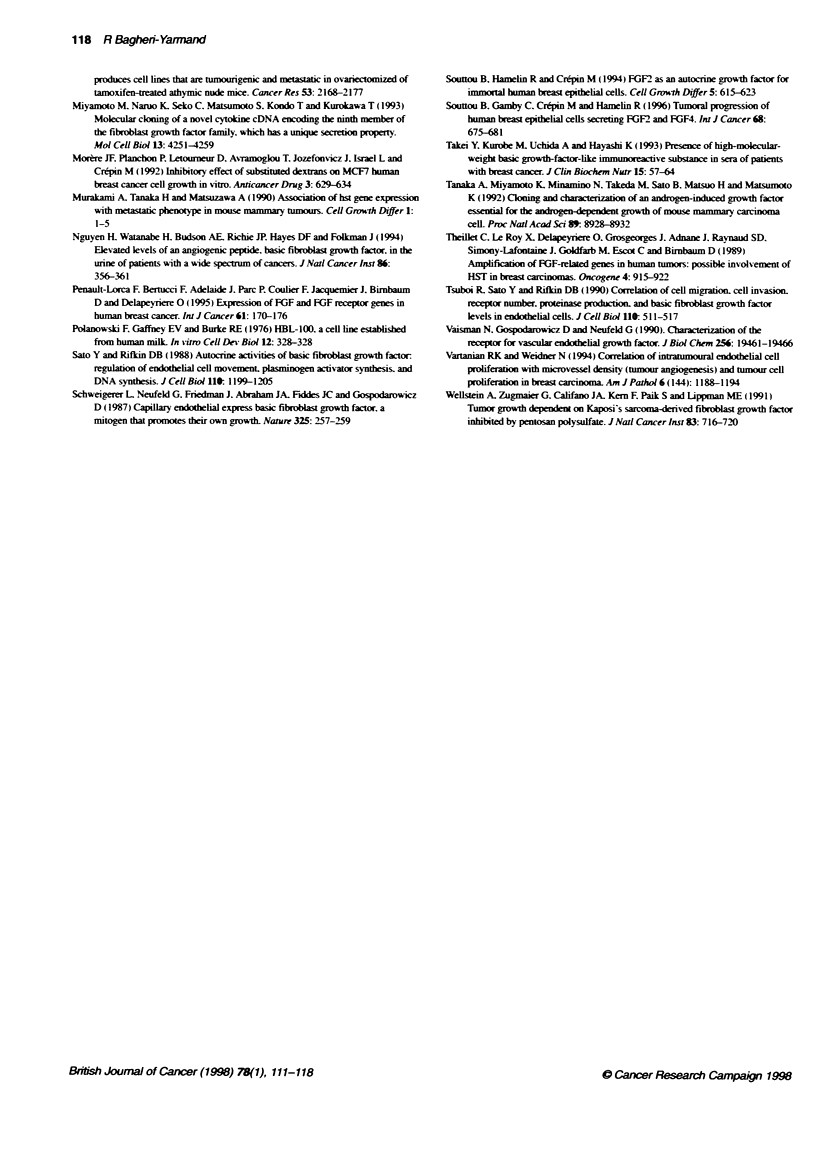


## References

[OCR_00902] Alroy J., Goyal V., Skutelsky E. (1987). Lectin histochemistry of mammalian endothelium.. Histochemistry.

[OCR_00907] Bagheri-Yarmand R., Bittoun P., Champion J., Letourneur D., Jozefonvicz J., Fermandjian S., Crépin M. (1994). Carboxymethyl benzylamide dextrans inhibit breast cell growth.. In Vitro Cell Dev Biol Anim.

[OCR_00911] Bagheri-Yarmand R., Liu J. F., Ledoux D., Morère J. F., Crépin M. (1997). Inhibition of human breast epithelial HBL100 cell proliferation by a dextran derivative (CMDB7): interference with the FGF2 autocrine loop [corrected].. Biochem Biophys Res Commun.

[OCR_00915] Basilico C., Moscatelli D. (1992). The FGF family of growth factors and oncogenes.. Adv Cancer Res.

[OCR_00924] Czubayko F., Smith R. V., Chung H. C., Wellstein A. (1994). Tumor growth and angiogenesis induced by a secreted binding protein for fibroblast growth factors.. J Biol Chem.

[OCR_00929] Dickson C., Smith R., Brookes S., Peters G. (1984). Tumorigenesis by mouse mammary tumor virus: proviral activation of a cellular gene in the common integration region int-2.. Cell.

[OCR_00934] Folkman J., Klagsbrun M. (1987). Angiogenic factors.. Science.

[OCR_00939] Gomm J. J., Smith J., Ryall G. K., Baillie R., Turnbull L., Coombes R. C. (1991). Localization of basic fibroblast growth factor and transforming growth factor beta 1 in the human mammary gland.. Cancer Res.

[OCR_00953] Gualandris A., Rusnati M., Belleri M., Nelli E. E., Bastaki M., Molinari-Tosatti M. P., Bonardi F., Parolini S., Albini A., Morbidelli L. (1996). Basic fibroblast growth factor overexpression in endothelial cells: an autocrine mechanism for angiogenesis and angioproliferative diseases.. Cell Growth Differ.

[OCR_00944] Gualandris A., Urbinati C., Rusnati M., Ziche M., Presta M. (1994). Interaction of high-molecular-weight basic fibroblast growth factor with endothelium: biological activity and intracellular fate of human recombinant M(r) 24,000 bFGF.. J Cell Physiol.

[OCR_00958] Hlatky L., Tsionou C., Hahnfeldt P., Coleman C. N. (1994). Mammary fibroblasts may influence breast tumor angiogenesis via hypoxia-induced vascular endothelial growth factor up-regulation and protein expression.. Cancer Res.

[OCR_00963] Hori A., Sasada R., Matsutani E., Naito K., Sakura Y., Fujita T., Kozai Y. (1991). Suppression of solid tumor growth by immunoneutralizing monoclonal antibody against human basic fibroblast growth factor.. Cancer Res.

[OCR_00969] Kim K. J., Li B., Winer J., Armanini M., Gillett N., Phillips H. S., Ferrara N. (1993). Inhibition of vascular endothelial growth factor-induced angiogenesis suppresses tumour growth in vivo.. Nature.

[OCR_00974] Klagsbrun M. (1989). The fibroblast growth factor family: structural and biological properties.. Prog Growth Factor Res.

[OCR_00978] Lebeau J., Le Chalony C., Prosperi M. T., Goubin G. (1991). Constitutive overexpression of a 89 kDa heat shock protein gene in the HBL100 human mammary cell line converted to a tumorigenic phenotype by the EJ/T24 Harvey-ras oncogene.. Oncogene.

[OCR_00984] Liu J., Bagheri-Yarmand R., Xia Y., Crépin M. (1997). Modulations of breast fibroblast and carcinoma cell interactions by a dextran derivative (CMDB7).. Anticancer Res.

[OCR_00991] McLeskey S. W., Kurebayashi J., Honig S. F., Zwiebel J., Lippman M. E., Dickson R. B., Kern F. G. (1993). Fibroblast growth factor 4 transfection of MCF-7 cells produces cell lines that are tumorigenic and metastatic in ovariectomized or tamoxifen-treated athymic nude mice.. Cancer Res.

[OCR_01000] Miyamoto M., Naruo K., Seko C., Matsumoto S., Kondo T., Kurokawa T. (1993). Molecular cloning of a novel cytokine cDNA encoding the ninth member of the fibroblast growth factor family, which has a unique secretion property.. Mol Cell Biol.

[OCR_01016] Nguyen M., Watanabe H., Budson A. E., Richie J. P., Hayes D. F., Folkman J. (1994). Elevated levels of an angiogenic peptide, basic fibroblast growth factor, in the urine of patients with a wide spectrum of cancers.. J Natl Cancer Inst.

[OCR_01022] Penault-Llorca F., Bertucci F., Adélaïde J., Parc P., Coulier F., Jacquemier J., Birnbaum D., deLapeyrière O. (1995). Expression of FGF and FGF receptor genes in human breast cancer.. Int J Cancer.

[OCR_01031] Sato Y., Rifkin D. B. (1988). Autocrine activities of basic fibroblast growth factor: regulation of endothelial cell movement, plasminogen activator synthesis, and DNA synthesis.. J Cell Biol.

[OCR_01036] Schweigerer L., Neufeld G., Friedman J., Abraham J. A., Fiddes J. C., Gospodarowicz D. (1987). Capillary endothelial cells express basic fibroblast growth factor, a mitogen that promotes their own growth.. Nature.

[OCR_01045] Souttou B., Gamby C., Crepin M., Hamelin R. (1996). Tumoral progression of human breast epithelial cells secreting FGF2 and FGF4.. Int J Cancer.

[OCR_01041] Souttou B., Hamelin R., Crépin M. (1994). FGF2 as an autocrine growth factor for immortal human breast epithelial cells.. Cell Growth Differ.

[OCR_01057] Tanaka A., Miyamoto K., Minamino N., Takeda M., Sato B., Matsuo H., Matsumoto K. (1992). Cloning and characterization of an androgen-induced growth factor essential for the androgen-dependent growth of mouse mammary carcinoma cells.. Proc Natl Acad Sci U S A.

[OCR_01065] Theillet C., Le Roy X., De Lapeyrière O., Grosgeorges J., Adnane J., Raynaud S. D., Simony-Lafontaine J., Goldfarb M., Escot C., Birnbaum D. (1989). Amplification of FGF-related genes in human tumors: possible involvement of HST in breast carcinomas.. Oncogene.

[OCR_01070] Tsuboi R., Sato Y., Rifkin D. B. (1990). Correlation of cell migration, cell invasion, receptor number, proteinase production, and basic fibroblast growth factor levels in endothelial cells.. J Cell Biol.

[OCR_01073] Vaisman N., Gospodarowicz D., Neufeld G. (1990). Characterization of the receptors for vascular endothelial growth factor.. J Biol Chem.

[OCR_01076] Vartanian R. K., Weidner N. (1994). Correlation of intratumoral endothelial cell proliferation with microvessel density (tumor angiogenesis) and tumor cell proliferation in breast carcinoma.. Am J Pathol.

[OCR_01081] Wellstein A., Zugmaier G., Califano J. A., Kern F., Paik S., Lippman M. E. (1991). Tumor growth dependent on Kaposi's sarcoma-derived fibroblast growth factor inhibited by pentosan polysulfate.. J Natl Cancer Inst.

